# Single-center experience in 127 adult patients, mono or dual artificial liver support therapy, in patients with acute liver failure

**DOI:** 10.3389/fmed.2023.1190067

**Published:** 2023-09-22

**Authors:** Ilhan Ocak

**Affiliations:** Department of Liver Transplant Critical Care Medicine, Memorial Sisli Hospital, Istanbul, Türkiye

**Keywords:** acute liver failure, dual supportive extracorporeal therapy, liver transplantation, mono supportive extracorporeal therapy, mortality

## Abstract

**Background:**

Acute liver failure (ALF) is a serious condition characterized by sudden liver dysfunction, jaundice and hepatic encephalopathy. Its mortality rate of approximately 80% underscores the urgent need for effective treatments. Supportive extracorporeal therapies (SET), which temporarily support liver function and remove toxins, have shown promise in improving outcomes in acute liver failure (ALF). The aim of this study was to compare the outcomes of dual supportive extracorporeal therapy (SET) and mono supportive extracorporeal therapy in patients with acute liver failure.

**Methods:**

A total of 127 patients with acute liver failure were included in this retrospective, single-center study. Of these, 62 patients received dual supportive extracorporeal therapy and 65 patients received mono supportive extracorporeal therapy. Primary endpoints were survival without the need for liver transplantation and mortality. Secondary endpoints included resolution of encephalopathy and normalization of International Normalized Ratio (INR).

**Results:**

In the dual supportive extracorporeal therapy group, 59.6% of patients survived without the need for liver transplantation, while 27.4% achieved recovery with liver transplantation. The mortality rate in this group was 12.9%. Significant regression of encephalopathy grade was observed in 87% of patients, and the 1 year mortality rate for liver transplant recipients was 10.7%. In the mono supportive extracorporeal therapy group, 61.5% of patients experienced a successful recovery without the need for liver transplantation, with a mortality rate of 29.2%. Significant improvement in the grade of encephalopathy was observed in 70.7% of patients.

**Conclusion:**

Both dual supportive extracorporeal therapy (CVVHDF and PE) and mono supportive extracorporeal therapy (PE) were associated with significant improvements in renal and hepatic biochemical parameters, blood ammonia levels, and neurological status in patients with acute liver failure associated with grade III-IV hepatic encephalopathy. In particular, dual support was associated with improved hemodynamic stability, lactic acidosis and acid–base balance. Survival in acute liver failure in our retrospective cohort using a protocolized approach to extracorporeal therapies is higher compared to previously published large ALF studies. This protocolized approach warrants further prospective studies.

## 1. Introduction

Acute liver failure (ALF) is characterized by the sudden onset of jaundice, impaired synthesis of liver proteins, and hepatic encephalopathy in individuals without a preexisting liver disorder. It is a grave and intricate condition that arises from the abrupt and extensive destruction of liver cells (1–3). Acute liver failure carries a grim prognosis, as it is associated with an approximate mortality rate of 80% (4). However, through enhanced comprehension of the underlying factors contributing to ALF, targeted supportive extracorporeal therapies (SET), advancements in intensive care practices, and the accessibility of liver transplantation (LT), the prognosis for ALF patients has significantly improved (5). Presently, among patients with ALF, over 43% manage to survive with SET without requiring a liver transplant, while approximately 28% do not survive the condition, and roughly 29% undergo a liver transplantation procedure. In addition, individuals who undergo a liver transplant exhibit a 1 year survival rate of around 60–90% (6). Acute liver failure is a rare condition, occurring in only 1–8 instances per million individuals, and it contributes to 6% of liver disease-related deaths and up to 7–8% of liver transplantations. The progression of cerebral swelling, sepsis, and failure of multiple organs are the primary factors leading to mortality (7–9).

Supportive extracorporeal therapy is a medical procedure that offers temporary and partial substitution of liver function, alongside the elimination of detrimental substances and provision of advantageous biological elements. This supportive extracorporeal therapy aims to facilitate the revitalization and functional recuperation of the patient’s liver cells (10, 11). Continuous venovenous hemodiafiltration (CVVHDF) and plasma exchange (PE) are used as mono or dual SET, both for bridge to liver transplant or Recovery in ALF patients (11–13).

This 15 years retrospective study aimed to evaluate supportive extracorporeal therapy (SET), including dual (CVVHDF and PE) and mono (PE) therapies, for 127 adults with acute liver failure.

### 1.1. Dual supportive extracorporeal therapy

Dual supportive extracorporeal therapy (SET) involves the concurrent use of plasma exchange and continuous venovenous hemodiafiltration. This treatment regimen entails performing one or two plasma exchange sessions per day while simultaneously continuing with continuous venovenous hemodiafiltration.

## 2. Materials and methods

### 2.1. Patients

A retrospective analysis of the medical records of 127 adult patients followed up in the Memorial Sisli Hospital Organ Transplant Center, Istanbul, Turkey, between January 2006 and December 2020 was included in this study. It consisted of two groups, 65 patients receiving mono SET-PE and 62 patients receiving dual SET-PE and CVVHDF (CRRT). The hospital performed an average of 100 liver transplants per year during this period, and all patients in the study were selected from those followed in the Intensive Care Unit. Patients also received conventional hepatic failure and hepatic encephalopathy (HE) medical treatment options.

Acute liver failure was characterized on the basis of the following conditions: (1) the absence of known chronic liver disease; (2) the presence of biochemical indicators of acute liver failure, such as elevated transaminase levels, within a period of less than 8 weeks; (3) the presence of liver-related coagulopathy, as indicated by a prothrombin time (PT) of 15 s or higher or an international normalized ratio (INR) of 1. 5 or higher that does not improve with vitamin K administration, together with clinical evidence of hepatic encephalopathy (HE), or a PT of 20 s or higher or an INR of 2.0 or higher, regardless of the presence of HE (14).

**Figure 1:**
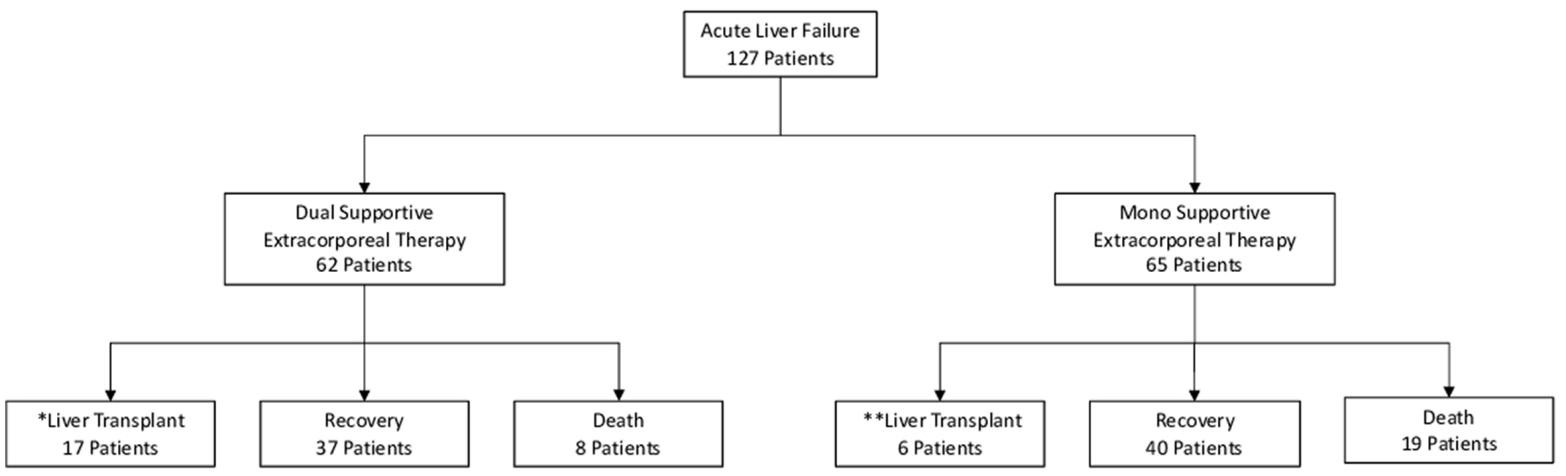
Flow diagram of patients in the study.

In patients with acute liver failure (ALF), the decision for liver transplantation was based on the End Stage Liver Disease Model-Na (MELD-Na) score. Mono SET with plasma exchange (PE) was considered for inclusion if the patient had high PT/INR levels or high PT/INR and ammonia levels (grade 3–4 encephalopaty). Dual SET with continuous venovenous hemodiafiltration (CVVHDF) and PE was included if the patient had high PT/INR and ammonia levels (grade 3–4 encephalopathy) or developed renal failure such as hepatorenal syndrome. The primary objective of these therapies was to serve as a bridge to liver transplantation or promote recovery. Patients who did not have ALF or did not receive supportive extracorporeal therapy were excluded from the study (Figure 1; Table 1).

**Table 1:** Data on acute liver failure patients monitored in the intensive care unit.

	Dual SET	Mono SET	Total
Demographics (*N*)			
Male	23	27	50
Female	39	38	77
Age (range)	40 (18–63)*	44 (21–76)*	42(18–76)*
ICU Scores			
APACHE II (range)	25 (20–28)*	22 (20–28)*	24(20–28)*
MELD-Na (range)	36.5 (32–40)*	32 (26–40)*	34(26–40)*
Etiology (*N*)			
Autoimmune hepatitis	5	N/A	5
Hepatitis-B	4	N/A	4
Paracetamol	10	21	31
Non-paracetamol drug	15	18	33
Toxication (mushrooms. Etc.)	12	24	36
Cause unknown	14	2	16
Surgical complication	2	N/A	2
Liver transplant patients (*N*)	17	6	23

*Median (minimum–maximum), (N), Number; N/A, not applicable. APACHE, acute physiology and chronic health evaluation; ICU, intensive care unit; MELD, model for end-stage liver disease, SET, supportive extracorporeal therapy.

### 2.2. SET protocol

We used the protocol published by Ocak (15) for the indication of mono or dual supportive extracorporeal therapy and liver transplantation. Our apheresis technicians and nurses performed the supportive extracorporeal treatment in our unit in accordance with this protocol. The grade of hepatic encephalopathy was assessed using the West Haven classification.

### 2.3. CVVHDF (CRRT) protocol

We employed a renal replacement device and hemodiafiltration kit manufactured by Fresenius Medical Care, based in Bad Homburg, Germany, for the purpose of conducting continuous venovenous hemodiafiltration. Throughout the day, we consistently administered continuous citrate-calcium anticoagulation, maintaining blood flow rates between 3 and 5 mL per kilogram per minute, along with dialysate flow rates ranging from 180 to 300 mL per kilogram per hour. The dialysate and replacement solutions employed encompassed multibic, multiplus, citrate-calcium dialysate, citrate-calcium dialysate-potassium 2 plus, and 4% sodium citrate.

### 2.4. PE protocol

Plasma exchange was conducted using a continuous renal replacement device in conjunction with the Fresenius plasma exchange Kit. The treatment was administered twice daily for a duration of 2–6 h per session. The flow rate for each session was calculated at 10–30 cc/min, and the volume of sessions was determined as 50 cc/kg. Additionally, 1.5 volumes of fresh frozen plasma were used to replace an equivalent volume without the use of anticoagulant treatment. The study encompassed the analysis of laboratory values and monitoring of vital signs both before and after the supportive extracorporeal therapy. It also examined the duration and frequency of the plasma exchange treatments.

### 2.5. Ethics approval

The retrospective study was approved by the Institutional Review Board of Memorial Sisli Hospital with the date 03/06/2022 and number 522/22. In light of the retrospective nature of the study, Informed consent was not required.

### 2.6. Statistical analyses

The statistical analysis was performed using the Statistical Package for Social Sciences version 26.0 (IBM Corp.; Armonk, New York, United States). To assess the normality of the study data, the Kolmogorov–Smirnov analysis was employed. For normally distributed data, the mean values were utilized, whereas non-normally distributed data was represented using the median (interquartile range). To compare the pre- and post-session laboratory values for supportive extracorporeal therapy, the Wilcoxon test was utilized. For this study, a significance level of less than 0.05 (*p* < 0.05) was considered statistically significant.

## 3. Results

Inclusion and exclusion criteria, demographic information, etiolgy, the Acute Physiology and Chronic Health Evaluation 2 score, the Model for End-stage Liver Disease-Na score, and the etiology were all documented when adult patients were admitted to the Intensive Care Unit (Table 1).

### 3.1. Dual and mono SET results

A group of 62 dual SET patients received dual supportive extracorporeal therapy for an average of 9 days. Among these patients, all 62 received plasma exchange treatments twice daily. The number of sessions varied from 6 to 36, with an average of 17.98. In addition, the average duration of continuous venovenous hemodiafiltration was 230 h (equivalent to 9.58 days), ranging from 71 to 434 h.

In the group of 65 mono SET patients, mono supportive extracorporeal therapy was administered for an average of 7 days. Among these patients, all 65 received twice daily plasma exchange treatments. The number of sessions ranged from 4 to 28, with an average of 14.11.

### 3.2. Laboratory values of before and after SET

Prior to the initial implementation of dual supportive extracorporeal therapy, the levels of total bilirubin in the serum of patients who underwent liver transplants were significantly higher compared to those patients who did not receive liver transplants. However, ALT and AST levels were substantially lower. Following the application of dual supportive extracorporeal therapy, the patients who did not undergo liver transplants exhibited notably better results in terms of serum ammonia, PT/INR, and total bilirubin measurements (Table 2). After the last dual SET, both patients with and without liver transplants showed significantly lower levels of total bilirubin, ALT, AST, ammonia, creatinine, and PT/INR compared to their initial levels before the dual SET. Additionally, there were significant improvements in blood gas parameters such as pH, bicarbonate, and lactate. The platelet count showed a significant increase, indicating better blood clotting. Moreover, there were improvements in mean arterial pressure (MAP) and heart rate, suggesting better cardiovascular stability. A significant decrease in the creatinine level also shows that it can prevent the negative effects of renal dysfunction (Table 3). After the last mono SET, the serum levels of total bilirubin, ALT, AST, ammonia, and PT/INR showed a significant decrease compared to the initial levels before mono SET in all patients. However, there was no significant improvement observed in the pH, bicarbonate, and lactate levels in the blood gas analysis. Furthermore, there was no significant improvement in the platelet count and creatinine level. Similarly, there was no significant improvement in the mean arterial pressure (MAP) and heart rate (Table 4). Due to the small number of patients who underwent liver transplantation and mono SET, biochemical comparisons were not made.

**Table 2:** Laboratory values in patients with acute liver failure with or without liver transplant before the start of dual supportive extracorporeal therapy (SET) and after the final of dual SET.

	Starting values* pre SET	Final values* post SET	*p* values
Dual SET liver transplant patients (17)**			
Aspartate Transaminase (IU/L)	1,621 (1101)	186 (82)	<0.01
Alanine Transaminase (IU/L)	1701 (1129)	197.5 (83.07)	<0.01
Lactate (mmol/L)	5.69 (1.14)	1.67 (0.2)	<0.01
Ammonia (μmol/L)	127 (27.5)	76 (15)	<0.01
Total Bilirubin (mg/dL)	19.01 (8.14)	5.9 (3.89)	<0.01
Prothrombin Time/International Normalized Ratio	3.34 (0.7)	1.7 (0.1)	<0.01
Without liver transplant patients (45)**			
Aspartate Transaminase (IU/L)	2,620 (1798)	179 (119.5)	<0.01
Alanine Transaminase (IU/L)	2,729 (1897)	191 (105.5)	<0.01
Lactate (mmol/L)	5.9 (2.5)	1.22 (0.60)	<0.01
Ammonia (μmol/L)	131 (45.5)	52 (15)	<0.01
Total Bilirubin (mg/dL)	8.1 (3.86)	2.2 (0.7)	<0.01
Prothrombin Time/International Normalized Ratio	3.3 (0.65)	1.29 (0.2)	<0.01

*Median (IQR) and **Number of patients. SET, supportive extracorporeal therapy.

**Table 3:** Dual supportive extracorporeal therapy (SET) laboratory values before the start of dual SET and after the final of dual SET.

Dual SET all patients	Starting values* pre SET	Final values* post SET	*p* values
Aspartate transaminase (IU/L)	2,301 (1416)	182 (109.21)	<0.01
Alanine transaminase (IU/L)	2,317 (1329)	195 (102.15)	<0.01
Lactate (mmol/L)	6.01 (1.46)	1.51 (0.7)	<0.01
Ammonia (μmol/L)	131 (36)	58 (23)	<0.01
Total bilirubin (mg/dL)	9.15 (8.55)	2.59 (2.1)	<0.01
Prothrombin time/International normalized ratio	3.5 (0.70)	1.34 (0.4)	<0.01
Ph	7.29 (0.1)	7.41	<0.01
Mean arterial pressure	56 (6)	66 (2)	<0.01
Creatinine	1.41 (0.5)	0.5 (0.2)	<0.01
Heart rate	122 (21)	94 (11)	<0.01
PaO_2_/FiO_2_	276 (34)	302.1 (20.19)	<0.01
Platelets	54 (22)	86 (27)	<0.01
Bicarbonate	16.40 (1.93)	22.8 (1)	<0.01

*Median (IQR). FiO_2_, fraction of inspired oxygen; Ph, potential of hydrogen; PaO_2_, partial pressure of oxygen; SET, supportive extracorporeal therapy.

**Table 4:** Mono supportive extracorporeal therapy (SET) laboratory values before the start of mono SET and after the final of mono SET.

Mono SET all patients	Starting values* pre SET	Final values* post SET	*p* values
Aspartate transaminase (IU/L	2,540 (1787)	185 (119.5)	<0.01
Alanine transaminase (IU/L)	2,748 (1936)	192 (107.5)	<0.01
Lactate (mmol/L)	5.8 (2.7)	1.23 (0.63)	<0.01
Ammonia (μmol/L)	131 (46)	55 (17)	<0.01
Total bilirubin (mg/dL)	8.1 (3.94)	2.1 (0.7)	<0.01
Prothrombin time/International normalized ratio	3.3 (0.76)	1.29 (0.15)	<0.01
Ph	7.30 (0.1)	7.31 (0.1)	<0.146
Mean arterial pressure	58 (6)	59 (2)	<0.132
Creatinine	1.19 (0.4)	1.18 (0.2)	<0.122
Heart rate	109 (20)	106 (12)	<0.09
PaO_2_/FiO_2_	264 (28.75)	255 (24)	<0.113
Platelets	56 (22.5)	58 (23)	<0.100
Bicarbonate	20.01 (2.48)	20.29 (1)	<0.105

*Median (Iqr). FiO_2_, fraction of inspired oxygen; Ph, potential of hydrogen; PaO_2_, partial pressure of oxygen; SET, supportive extracorporeal therapy.

### 3.3. Comparison of dual SET and mono SET results

Comparing the effects of mono SET and dual SET, both therapy approaches resulted in significant improvements in various indicators of organ recovery. In the case of dual SET, which involved patients with and without liver transplants, there were notable reductions in total bilirubin, ALT, AST, ammonia, creatinine, and PT/INR levels compared to their initial values. These reductions may represent clearance effects of both therapies or improvement intrinsic organ function. Additionally, blood gas parameters such as pH, bicarbonate, and lactate showed significant improvements, indicating improved acid–base balance. The increase in platelet count suggests improved blood clotting, while the improvements in mean arterial pressure (MAP) and heart rate point towards better cardiovascular stability. On the other hand, mono SET led to significant decreases in total bilirubin, ALT, AST, ammonia, and PT/INR levels in all patients. These reductions may represent clearance effects of mono SET or improvement intrinsic organ function, similar to Dual SET. However, the blood gas analysis did not show significant improvements in pH, bicarbonate, and lactate levels, suggesting that acid–base balance might not have been as effectively regulated by Mono SET. Additionally, there were no significant improvements in platelet count and creatinine levels, suggesting that blood clotting and kidney function may not have been benefited significantly by this treatment approach. Similarly, there were no significant improvements in mean arterial pressure (MAP) and heart rate, indicating that cardiovascular stability might not have been as positively influenced by mono SET compared to dual SET. In summary, while both mono SET and dual SET showed improvements in liver function, Dual SET appeared to have more comprehensive effects, including better acid–base balance, improved blood clotting, and cardiovascular stability.

### 3.4. Adverse effects of SET

During the use of dual supportive extracorporeal therapy, hypernatremia developed in 3 patients, metabolic alkalosis in 3 patients, and hypocalcemia in 2 patients. Nevertheless, the introduction of hemodiafiltration as part of the supportive extracorporeal therapy provided beneficial results by alleviating these conditions. In addition, five individuals experienced a superficial allergic reaction due to the use of fresh frozen plasma during plasma exchange. As a precaution, the procedure was temporarily halted and a new batch of fresh frozen plasma was used. After resolution of the allergic reaction, the procedure was continued. During mono supportive extracorporeal therapy, hypervolemia developed in 4 patients, hypernatremia in 5 patients, metabolic alkalosis in 6 patients, and hypocalcemia in 3 patients during plasma exchange. However, appropriate supportive care was provided and these conditions improved. In addition, six subjects experienced a superficial allergic reaction due to the use of fresh frozen plasma during plasma exchange. Again, the procedure was temporarily suspended. After resolution of the allergic reaction, the procedure was resumed.

Despite the use of local citrate in CVVHDF application, side effects such as electrolyte disturbances did not develop due to balanced antidote (calcium) and dialysate solutions. This was controlled by monitoring electrolytes and blood gases every 2 hours. Macro- and micronutrient deficiencies also did not develop.

### 3.5. Mortality

Of the 62 patients who received dual supportive extracorporeal therapy (SET), 37 patients (59.6%) survived without requiring liver transplantation. In addition, 17 patients (27.4%) survived but required liver transplantation. Mortality rate in this group was 12.9% (8 patients), with all deaths occurring within the first 18 days of dual SET. In addition, patients who underwent liver transplantation had a 1 year mortality rate of 10.7%. In contrast, 40 (61.5%) of 65 patients who underwent mono supported extracorporeal therapy had a successful recovery, and 6 patients (9.2%) survived but required liver transplantation. However, mortality rate was higher in this group at 29.2% (19 patients), with all deaths occurring within the first 14 days of mono SET.

## 4. Discussion

### 4.1. Discussion of dual SET and mono SET findings

In this study, we evaluated two groups of patients with acute liver failure who were followed in the intensive care unit, consisting of 62 patients receiving dual supportive extracorporeal therapy and 65 patients who received mono supportive extracorporeal therapy. When comparing the outcomes of patients who received dual supportive extracorporeal therapy (SET) with those who received mono-supportive extracorporeal therapy, significant differences were observed. In the case of dual SET, out of 62 patients, a majority of 37 individuals (59.6%) were able to survive without the need for liver transplantation. Seventeen (27.4%) patients recovered with liver transplantation. However, it should be noted that the mortality rate for this group was 12.9% (8 patients). Of note, all deaths occurred within the first 18 days of dual SET. A remarkable 87% resolution or reduction in the grade of encephalopathy was also noted. In addition, the 1 year mortality rate for patients who received a liver transplant was 10.7%. Among the group of 65 patients who received mono-SET, a remarkable proportion of 40 individuals (equivalent to 61.5%) experienced a successful recovery without the need for liver transplantation. In addition, 6 patients (9.2%) achieved recovery with the use of liver transplantation. A remarkable 70.7% recovery in the grade of encephalopathy was also noted. However, the mortality rate for this group was relatively higher at 29.2% (19 patients). When comparing the two regimens, dual SET (11.3%) resulted in a lower mortality compared to mono SET (29.2%). However, the rate of successful recovery without the need for liver transplantation was similar in mono SET (61.5%) and dual-SET (59.6%). There was significant regression and improvement in all laboratory values in 8 patients who died in the dual SET group of 62 patients in this study. However, we found that the grade of encephalopathy could not be permanently regressed and the INR could not be permanently normalized. Although these patients survived for approximately 18 days, liver transplantation could not be performed because a suitable donor could not be found. We found that in 37 adult patients who recovered clinically, the encephalopathy grade regressed to 1 and below, while the INR was permanently normalized. In addition, mean arterial pressure increased significantly, vital signs stabilized, and fluid, electrolyte, and acid–base balance were preserved. In this group, liver transplantation was performed in 17 patients who did not have sufficient improvement in encephalopathy and continuous normalization of INR. However, bridge-to-transplant was performed with dual SET. Recovery was achieved by liver transplantation. In the mono SET group, which consisted of 65 patients, 19 patients died, although there was a significant decrease in all liver laboratory values, there was no significant improvement in renal laboratory values, hemodynamic parameters, and platelet count. In addition, it was found that the grade of encephalopathy was not permanently reduced and the INR was not permanently normalized in the patients who died in the mono SET group. Although these patients survived up to 14 days, liver transplantation could not be performed because a suitable donor could not be found. However, 40 clinically recovered adult patients in this group also improved to grade 1 or less encephalopathy and achieved sustained normalization of INR. In this group, liver transplantation was performed in 6 patients who did not have sufficient improvement in encephalopathy and continuous normalization of INR. In addition, although drug toxicity was the predominant etiology in the mono SET group, there was a similar recovery in the dual SET group. The mortality rate was found to be higher in the mono SET group (mortality rate 29.2%) compared to the dual SET group (mortality rate 12.9%). The high mortality rate may be due to the insufficient effect of Mono SET on non-hepatic organs and hemodynamics, as well as the lack of suitable donors for liver transplantation in this group. There was no significant difference between patients who died and those who survived in terms of the scales used to calculate the severity of liver disease (MELD-Na, APACHE 2 scores) (16).

### 4.2. Comparison of dual SET and mono SET findings

When we compared the groups in our study, the side effects in both groups were mostly due to the use of fresh frozen plasma (FFP). Reversibility was achieved with supportive care. In both groups, patients without permanent regression of encephalopathy grade and INR stability either underwent liver transplantation or died. In addition, dual SET had a favorable effect on the improvement of hemodynamic stability and biochemical parameters. There was also a significant improvement in cerebral function in the Dual SET group. This effect may be associated with the observed reduction in mortality and morbidity. Adverse effects in these two groups were associated with the use of FFP for replacement.

Recent studies have shown that dual liver support therapies (PE + CRRT) in adult (17–19) and pediatric (12, 15, 16, 20). ALF patients improve hepatic biochemical parameters, neurological status, and hemodynamic stability. In addition, they have been found to provide a bridge to liver transplantation. Similar to these studies, neurological status and hemodynamic stability improved in our study. Regression was observed in encephalopathy grade. Also bridge to transplantation was performed. In our study, the mortality rate was 12.9% and the liver transplantation rate was 27.4%.

In recent studies by Maiwall et al. (21, 22) (mortality rate 25%), and Larsen et al. (7, 23) (mortality rate 41.3%) in ALF patients, it was shown that mono liver support therapies (PE) improved liver biochemical parameters and neurological status. In addition, they showed that they provided a bridge to transplantation. In our study, similar to these studies, neurological status was improved. Also provided a bridge to transplantation. In our study, the mortality rate was 29.2% and the liver transplantation rate was 9.2%. It should be noted that at the end of dual or mono SET, accessibility to liver transplantation has a significant positive effect on survival.

### 4.3. Discussion of the mechanism of action of dual SET and mono SET

Acute liver failure is a catastrophic, complex pathophysiological process that can lead to rapid death in MOF. The primary goal of an ideal treatment should be to stop the cascade leading to this devastating process by removing circulating blood (24). Due to the nature of the disease, plasma products used to correct coagulopathy increase protein load and exacerbate hyperammonemia (24, 25). Ammonia is involved in the pathogenesis of central nervous system toxicity in ALF, and high ammonia levels have been shown to be a poor prognostic factor for herniation and cerebral edema. One of the most effective methods of extracorporeal therapy to reduce ammonia is CRRT (26–28). Therefore, the use of dual SET (PE + CRRT) is reasonable and may provide a successful bridge to liver transplantation (29, 30). Plasma exchange (mono SET) removes plasma cytokines and mediators of the systemic inflammatory cascade and provides repeat factors synthesized by the liver. Plasma exchange can be performed in patients who are in poor condition and can be used as supportive therapy for the patient until spontaneous recovery occurs or liver transplantation is possible. Plasma exchange is an extracorporeal treatment modality used in acute liver failure (31). High volume plasma exchange is recommended in the American Society for Apheresis (ASFA) guidelines as Category I and Grade 1A in the management of acute liver failure (32). Plasma exchange can be used as a supportive therapy until spontaneous recovery or liver transplantation.

### 4.4. Hypothesis

Dual supportive extracorporeal therapy (SET) is associated with lower mortality and improved outcomes compared with mono supportive extracorporeal therapy in patients with acute liver failure. The results of our study showed significant differences in outcomes between patients who received dual SET and those who received mono SET for the treatment of acute liver failure. The dual SET group had a lower mortality rate (12.9%) compared to the mono SET group (29.2%). In addition, a higher proportion of patients in the dual SET group recovered without the need for liver transplantation (59.6% vs. 61.5%). The observed lower mortality rate in the dual SET group suggests that the combination of supportive therapies used in this approach may have a synergistic effect in improving patient outcomes. The dual SET group also showed a remarkable resolution or reduction in the grade of encephalopathy (87%), indicating improved neurological function. In contrast, the mono SET group had a lower rate of improvement in encephalopathy (70.7%), which may have contributed to the higher mortality rate observed in this group.

In addition, the dual SET group had significant improvements in laboratory values, mean arterial pressure, vital signs, and fluid, electrolyte, and acid–base balance. These findings suggest that dual SET may have a more comprehensive effect on multiple organ systems, leading to improved hemodynamics and overall patient stability.

It is worth noting that both groups experienced limitations in performing liver transplantation due to the unavailability of suitable donors. However, in cases where liver transplantation was performed, the 1 year mortality rate was relatively low (10.7% for dual-SET and 9.2% for mono-SET), indicating the efficacy of transplantation in improving long-term survival.

The results of our study support the hypothesis that dual supportive extracorporeal therapy is associated with lower mortality and improved outcomes compared with mono supportive extracorporeal therapy in patients with acute liver failure. The combination of supportive therapies in dual SET appears to have a synergistic effect, resulting in better neurological function, improved hemodynamics and overall patient stability.

### 4.5. Limitations

Single center, retrospective nonrandomized study. Outcomes were not statistically compared between intervention groups due to limited number an variability in transplantation rates in both groups. Inferences in difference in outcomes between therapies are meant to be speculative and hypothesis generating. However survival outcomes from our center are higher than previously published studies.

## 5. Conclusion

Both dual supportive extracorporeal therapy (CVVHDF and PE) and mono supportive extracorporeal therapy (PE) were associated with significant improvements in renal and hepatic biochemical parameters, blood ammonia levels, and neurological status in patients with acute liver failure associated with grade III–IV hepatic encephalopathy. Dual supportive in particular was associated with improved hemodynamic stability, lactic acidosis and acid base balance. Survival in ALF in our retrospective cohort using a protocolized approach to extracorporeal therapies is higher compared to previously published large ALF studies. This protocolized approach warrant further prospective studies.

## Data availability statement

The original contributions presented in the study are included in the article/supplementary material, further inquiries can be directed to the corresponding author.

## Ethics statement

The retrospective study was approved by the Institutional Review Board of Memorial Sisli Hospital with the date 03/06/2022 and number 520/22.

## Author contributions

IO: conceptualization, data curation, formal analysis, funding acquisition, investigation, methodology, project administration, resources, software, supervision, validation, visualization, writing – original draft, and writing – review and editing.

## Abbreviations

ALF: Acute liver failure
ALT: Alanine transaminase
AST: Aspartate transaminase
CRRT: Continuous renal replacement therapy
CVVHDF: Continuous venovenous hemodiafiltration
ICU: Intensive care unit
INR: International normalized ratio
LT: Liver transplantation
MAP: Mean arterial pressure
MOF: Multiorgan failure
PE: Plasma exchange
PT: Prothrombin time
SET: supportive extracorporeal therapy
